# Integration of third generation biofuels with bio-electrochemical systems: Current status and future perspective

**DOI:** 10.3389/fpls.2023.1081108

**Published:** 2023-02-10

**Authors:** Amitap Khandelwal, Meenu Chhabra, Piet N. L. Lens

**Affiliations:** ^1^ Ryan Institute, School of Natural Sciences, University of Galway, Galway, Ireland; ^2^ Environmental Biotechnology Lab, Department of Biosciences & Bioengineering, Indian Institute of Technology, Jodhpur, India

**Keywords:** algae biomass, photobioreactor, microbial fuel cell, biofuels, bioelectricity

## Abstract

Biofuels hold particular promise as these can replace fossil fuels. Algae, in particular, are envisioned as a sustainable source of third-generation biofuels. Algae also produce several low volume high-value products, which enhance their prospects of use in a biorefinery. Bio-electrochemical systems such as microbial fuel cell (MFC) can be used for algae cultivation and bioelectricity production. MFCs find applications in wastewater treatment, CO_2_ sequestration, heavy metal removal and bio-remediation. Oxidation of electron donor by microbial catalysts in the anodic chamber gives electrons (reducing the anode), CO_2,_ and electrical energy. The electron acceptor at the cathode can be oxygen/NO_3_^-^/NO_2_^-^/metal ions. However, the need for a continuous supply of terminal electron acceptor in the cathode can be eliminated by growing algae in the cathodic chamber, as they produce enough oxygen through photosynthesis. On the other hand, conventional algae cultivation systems require periodic oxygen quenching, which involves further energy consumption and adds cost to the process. Therefore, the integration of algae cultivation and MFC technology can eliminate the need of oxygen quenching and external aeration in the MFC system and thus make the overall process sustainable and a net energy producer. In addition to this, the CO_2_ gas produced in the anodic chamber can promote the algal growth in the cathodic chamber. Hence, the energy and cost invested for CO_2_ transportation in an open pond system can be saved. In this context, the present review outlines the bottlenecks of first- and second-generation biofuels along with the conventional algae cultivation systems such as open ponds and photobioreactors. Furthermore, it discusses about the process sustainability and efficiency of integrating algae cultivation with MFC technology in detail.

## 1 Introduction

Biofuels hold tremendous promise in providing energy security for the future. These are renewable, environment friendly, usable in existing engines, blendable with diesel, and available in liquid, gas, and solid form. Biofuels have been explored extensively during the last few decades (Chowdhury and Loganathan, 2019). Based on the original raw material for biofuel production, biofuels are categorized as first, second, and third generation. The first generation (1G) biofuels involve the use of food-based biomass feedstock like sugarcane, potato, corn, beet, sunflower, rapeseed and so forth. The use of 1G biofuels trigger the food versus fuel debate and is often limited by the availability of agricultural land. The direct use of food crops is highly unsustainable, particularly in highly populated developing countries. The 2G biofuels are derived from inedible portions of the plant and non-food items such as ligno-cellulosic wastes, waste cooking oil and carbon rich industry waste. (Chowdhury and Loganathan, 2019). The production of 2G biofuels is limited by the need to pretreat biomass, remove inhibitors, develop an enzymatic cocktail for hydrolysis, and develop an efficient fermenting strain.

The bottlenecks associated with the 1G and 2G biofuels switched researcher’s focus towards the evolution of 3G biofuels (Brennan and Owende, 2010). The third-generation biofuels are obtained from microalgae biomass. This generation of biofuels circumvents some of the problems associated with 1G and 2G biofuels and is relatively sustainable (Nigam and Singh, 2011). Algae is a source of several other high-value low-volume products that enable their use in a biorefinery (Chisti, 2007). The 3G biofuels hold several advantages over 1G and 2G biofuels, such as shorter harvesting cycle, higher growth rate, and higher oil production rate (Schenk et al., 2008). Algae cultivation does not depend on agricultural land eliminating the food versus fuel issue (Scott et al., 2010). Estimates show that a bio-oil productivity of 10000 L/hectare/year of bio-oil can be obtained from microalgae (Alam et al., 2015).

Algae are classified into two major categories based on their external morphology, i.e. microalgae and macroalgae. Brown and red algae along with green seaweed are prominent examples of macroalgae, whereas microalgae include *Chlorella, Spirulina* and other green algae (Demirbas, 2010). The microalgae are superior to macroalgae in terms of oil content, microscopic cell size, and higher growth rate. Algae biomass can be converted to bioethanol, biodiesel, biomethane, biohydrogen, biochar and some value added pigments or other value added products (Kumar et al., 2020).

### 1.1 Biodiesel

Biodiesel is mainly derived from the intracellular lipids of oleaginous microalgae. Algal lipids consist of triglycerides (TAG) along with mono and diglycerides and free fatty acids. Stearic acid, palmitic acid, and oleic acid are the predominant fatty acids types in the algal lipids (Tripathi et al., 2015). Algae biomass displays a variable amount of lipid content depending on the strain type and cultivation condition. For example, the lipid content of *Chlorella vulgaris* varies from 11% to 43% (Mitra et al., 2012). Enamala et al. (2018) reviewed several studies and found that the lipid content varies from 2.4% to 62% of dry algal cell weight (Enamala et al., 2018).

Algal lipids are converted to biodiesel through a catalytic transesterification reaction between triacylglycerols and methanol. The transesterification process results in fatty acid methyl ester (FAME) and glycerol (Kumar et al., 2020). The purified FAMEs are known as biodiesel. The lipid composition, such as the percentage of saturated fatty acids, affects the fuel properties. The algal oil contains more unsaturated fatty acids than saturated ones, which improves cold flow and make it a suitable feedstock (Demirbaş, 2009; Tripathi et al., 2015). However, it also triggers the production of hydroperoxide and insoluble substances which collectively lead to choking of the filter (Kumar and Thakur, 2018).

### 1.2 Biomethane

Algae biomass or leftover algal biomass after lipid extraction (Lipid extracted algae) produces biogas when subjected to anaerobic digestion. This biogas is composed of CH_4_ (50-70%) and CO_2_ (30-50%) (Kumar et al., 2020). The algal biomass can generate 0.024 –0.6 L CH_4_/g VS (volatile solid) or 0.2 –0.4 m^3^ CH_4_/kg biomass. The biogas yields vary from one species to another and depend on process conditions (Milledge et al., 2019; Rabii et al., 2019). The factors affecting biogas production include algae cell wall composition, process temperature, C/N ratio, biomass loading rate and reactor configuration (Sialve et al., 2009; McKennedy and Sherlock, 2015; Barbot et al., 2016). The biogas production process when integrated with other bioenergy processes adds value and makes it sustainable (Cesaro and Belgiorno, 2015).

### 1.3 Biochar

Biochar is produced through hydrothermal carbonization (HTC) of dry biomass (Gollakota et al., 2018). Algae based biochar has a high cation exchange capacity, lesser carbon proportion, and lesser surface area than lignocellulosic biomass based biochar (Michalak et al., 2019). Algae based biochar has higher yield compared to other feedstocks and the yield ranges from 8.1% to 64.2% of dry biomass (Yu et al., 2017; Michalak et al., 2019). The high ash content blocks micropores resulting in a low active surface area (Leng et al., 2021). Algae biochar also possesses several functional groups making it suitable for the remediation of inorganic and organic contaminants from wastewater (Kumar et al., 2020).

### 1.4 Bioethanol

Algal biomass can ferment to bioethanol under anaerobic conditions. The process is mediated by yeast, bacteria and/or fungi (Minh and Hanh, 2012; Robak and Balcerek, 2018). The algae biomass contains several polymers like mannitol and agar (Kostas et al., 2016; Offei et al., 2018). Brown algae are rich in carbohydrates such as alginate, mannitol, laminarin, glucose, fucoidan, and cellulose (Ale and Meyer, 2013). Similarly, red algae have a diverse range of hydrolysable polymers, which can be converted to ethanol (Behera et al., 2015).

### 1.5 Other value-added products

Intact algae biomass or algae products find applications in industries such as food, pharmaceutical, healthcare and cosmetics. Algal species such as *Spirulina* and *Chlorella* serve as a food supplement and source of protein (Kumar et al., 2020). Algae produce pigments like carotenoids, phycocyanin and chlorophyll (Barkia et al., 2019). Carotenoids such as zeaxanthin, α-carotene, β-carotene, and lutein are antioxidants and have anticancer properties (Dickinson et al., 2017; Matos, 2017). The polyunsaturated fatty acids (PUFA) derived from algae serve as food supplements (Lee, 2013). In addition to this, some unconventional value-added products such as ubiquinone coenzyme Q_10_, ubiquinol, cannabinoids, anandamids, hoshinolactum, dolastatins, endotoxins and several therapeutic substances can be obtained from algae (Abu-ghosh et al., 2021; Hans et al., 2021; Mondal et al., 2020).

## 2 Oleaginous algae

Algae are classified in nine groups, namely cyanobacteria (Cyanophyceae), diatoms (Bacillariophyceae), brown algae (Phaeophyceae), yellow-green algae (Xanthophyceae), red algae (Rhodophyceae), green algae (Chlorophyceae), golden algae (Chrysophyceae), “picoplankton” (Prasinophyceae and Eustigmatophyceae) and dinoflagellates (Dinophyceae) (Neto et al., 2019). Microalgae such as *Chlorella, Spirulina, Haematococcus* and *Dunaliella* are grown commercially with a production level of several 100 tons annually (Neto et al., 2019). These algae are a rich source of protein, carbohydrate, and lipid (**Table 1**). However, the chemical constitution of a microalgae cell can differ according to the species, strain and cultivation conditions (Lim et al., 2021). For example, it is reported that microalgae species such as *Trachydiscus* and *Nanochloropsis* are unable to produce carbohydrates (Hildebrand et al., 2013). Similarly, *Dunaliella tertiolecta* ATCC 30929 can produce lipids up to 74% (w/w) (Takagi et al., 2006), while *Chlorella vulgaris* CCAP 211/11B majorly produces carbohydrates (55% w/w) (Illman et al., 2000). The selection of a suitable strain for maximizing the biofuel production is crucial for the downstream processes (Debnath et al., 2021; Lim et al., 2021).

**Table 1:** Biochemical composition of different microalgae species.

Microalgae species	Lipid (%)	Protein (%)	Carbohydrate (%)	Reference
*Scenedesmus obliquus*	30-50	10-45	20-40	Debnath et al. (2021)
*Chlorella* sp. FC2IITG	15-54	22-40	18-46	Muthuraj et al. (2013)
*Chlorella vulgaris*	14-22	12-17	51-58	De Farias Silva et al. (2019)
*Chlorella sorokiniana*	22-24	40.5	26.8	Debnath et al. (2021)
*Chlamydomonas reinhardtii*	21	17	48	Hossain and Mahlia (2019)
*Dunaliella tertiolecta*	18-23.5	8.3-31.3	46.5-50.6	Efremenko et al. (2012)
*Nostoc commune*	22	20-43	34-56	Debnath et al. (2021)
*Rhodomonas* sp.	15	74	9	Hossain and Mahlia (2019)
*Spirogyra* sp.	16	55	20	Hossain and Mahlia (2019)
*Spirulina platensis*	4-9	46-63	8-14	De Farias Silva et al. (2019)

The presence of saturated and unsaturated fatty acids and their amounts also affect their suitability for employing them as engine oil. In a study, 7 freshwater microalgae species were compared by the presence of fatty acids. It was discovered that the C_16:2_, C_16:3_ and C_20:5_, C_16:4_ and C_18:4_, and C_18:4_ and C_22:6_ are only produced in *Nannochloropsis* sp., *Ankistrodesmus* sp., and *Isochrysis* sp., respectively (Thomas et al., 1984). Studies have confirmed that microalgal lipids are high in energy rich fatty acids and suitable for biofuel production (Steen et al., 2010). Furthermore, researchers around the globe have succeeded in developing strategies to improve lipid productivity from microalgae spp. In this context, growing microalgae in stress conditions such as nitrogen limitation has been shown very effective for some species (Levasseur et al., 2020). Recently, the development of genetic engineering tools and omics technologies have significantly improved lipid productivities in many strains (Muñoz et al., 2021).

## 3 Modes of algae cultivation

### 3.1 Open cultivation systems

#### 3.1.1 Open unagitated ponds

Unagitated and shallow open ponds require little effort for algae cultivation on a large scale. Natural water bodies having 50 cm depth are ideal for this kind of cultivation. The disadvantages associated with such systems include frequent contamination, slower diffusion of nutrients, and the formation of algal bloom (Chew et al., 2018).

#### 3.1.2 Circular ponds

Circular ponds are similar to unagitated open ponds except that they are equipped with a stirring unit. The mixing in circular ponds is enabled by a rotating shaft which moves in axial direction in order to create a homogenous mixing of nutrients (Ting et al., 2017). The circular ponds are also prone to contamination.

#### 3.1.3 Raceway ponds

Raceway ponds are extensively used for the commercial production of algae biomass. Raceway ponds have a race track type design and can have a single channel or multiple channels (Ting et al., 2017). Paddle wheels in these systems ensure mixing and homogenous suspension of algae cells.

### 3.2 Closed cultivation systems

#### 3.2.1 Horizontal tube photo-bioreactor

The horizontal tube photo-bioreactor (PBR) has long horizontal tubes arranged as panels, walls or helices (Chew et al., 2018). Mixing is achieved through a centrifugal pump (Klinthong et al., 2015). The reactors can run using either natural or artificial light. The limitation is the requirement of large surface areas.

#### 3.2.2 Vertical tube PBRs

Vertical tube PBRs, such as airlift and bubble column PBRs has an air sparger at the bottom of the reactor enabling mixing, nutrient, and gas exchange. The liquid flow in a bubble column reactor is triggered by the air bubbles produced at the bottom of the vessel. The high surface area to volume ratio of bubbles allows efficient gas exchange. An airlift reactor contains two interconnected regions, namely, dark and illuminated zones. Air bubbles lift the liquid from dark to light zones, leading to homogenous mixing of nutrients and fluids between the two zones. Vertical tube type PBRs offer homogenous mixing, low shear stress on cells, high photosynthetic efficiency, and high algal productivities (Chew et al., 2018).

#### 3.2.3 Flat panel PBR

Flat panel PBRs consist of two transparent plates arranged as rectangular box. The light source orientation ensures equal light intensity at all positions of the reactor. The air sparger and pump enables mixing and gas exchange (Klinthong et al., 2015). These systems have a high surface area to volume ratio, suitable design for scaling up, and low level of oxygen retention inside the reactor (Ting et al., 2017).

#### 3.2.4 Continuous stirred tank PBR

Continuous stirred tank reactor (CSTR) is similar to conventional CSTR bioreactors except for the presence of an external light source. These systems offer lower productivities due to inefficient light penetration and low surface area to volume ratio (Chew et al., 2018).

## 4 Integrating algae cultivation with bio-electrochemical systems

The main focus of all the commercial industries dealing with third generation biofuels is to optimize and develop efficient and cost-effective approaches for maximizing the algal biomass production. However, the existing algae cultivation strategies have several drawbacks which need to be addressed in order to commercialize the third-generation biofuels (**Table 2**). The major drawbacks associated with open ponds are that they are prone to contamination and evaporation losses. On the other hand, closed algae cultivation systems are highly expensive and often require oxygen quenching (**Table 2**).

**Table 2:** Pros and cons associated with conventional algae cultivation systems (Ting et al., 2017; Chew et al., 2018).

Cultivation system		Pros	Cons
Open	Open ponds	•Easy to build handle •Ideal for mass production at relatively affordable price	•Evaporation losses & prone to contamination •Requirement of large area & CO_2_ transportation from source to cultivation site
Closed	Tubular	•Ideal for outdoor cultivation •Temperature can be controlled •High biomass production	•Requirement of O_2_ quenching due to high DO concentration •Shading effect •Not suitable for scale-up processes
	Flat panel	•High algal growth rate •Comparatively lower O_2_ storage •High amount of light per unit area	•Difficulty in controlling temperature •Complexity in scale-up •Shading effect
	Continuous stir tank	•Better biomass yield due to good mixing •Minimum shading effect	•High cost associated with scale-up processes •Requirement of O_2_ quenching

**Figure 1:**
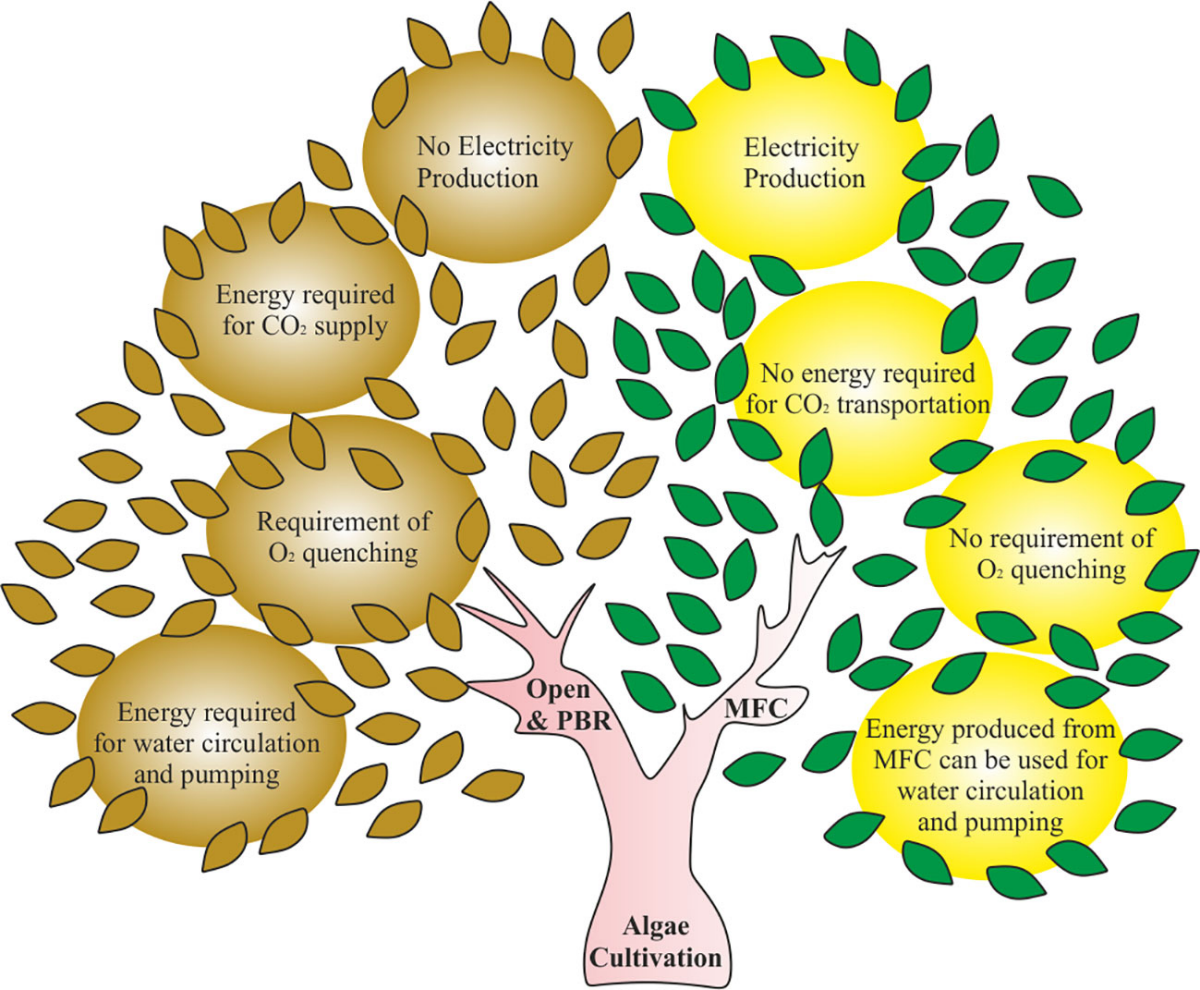
Illustration showing the comparison between conventional algae cultivation systems and microbial fuel cell based algae cultivation. PBR, Photobioreactor and MFC, Microbial fuel cell.

Bio-electrochemical systems such as microbial fuel cell (MFC) can be used for algae cultivation and bioelectricity production as they offer advantages over conventional algae cultivation systems. MFCs find applications in wastewater treatment, CO_2_ sequestration, heavy metal removal, and bio-remediation. (Zhang et al., 2011). A typical MFC consists of an anode and cathode placed in two chambers separated by an ion-exchange membrane. Oxidation of electron donor by microbial catalysts in the anodic chamber gives electrons (reducing anode), CO_2,_ and electrical energy (Khandelwal et al., 2022; Neethu et al., 2022). The electrons flow through the external circuit to be captured by the terminal electron acceptor present at the cathode (Trapero et al., 2017). The anode and cathode chamber have differences in redox potential, which is often maintained with the help of the ion-exchange membrane. The detailed description of MFCs can be found in the sections below. The electron acceptor at the cathode can be oxygen/NO_3_^-^/NO_2_^-^/metal ions. However, the need for a continuous supply of terminal electron acceptor in the cathode can be eliminated by growing algae in the cathodic chamber, as it produces enough oxygen through photosynthesis (Khandelwal et al., 2018). On the other hand, the conventional algae cultivation systems require periodic oxygen quenching, which involves further energy consumption and adds cost to the process. Therefore, the integration of algae cultivation and MFC technology can eliminate the need of oxygen quenching and external aeration in the MFC system and make the overall process sustainable and net energy producer. In addition to this, the CO_2_ gas produced in the anodic chamber can promote the algal growth in the cathodic chamber. Hence, the energy and cost invested for CO_2_ transportation in an open pond system can be saved (**Figure 1**).

### 4.1 MFC principles and components

**Figure 2:**
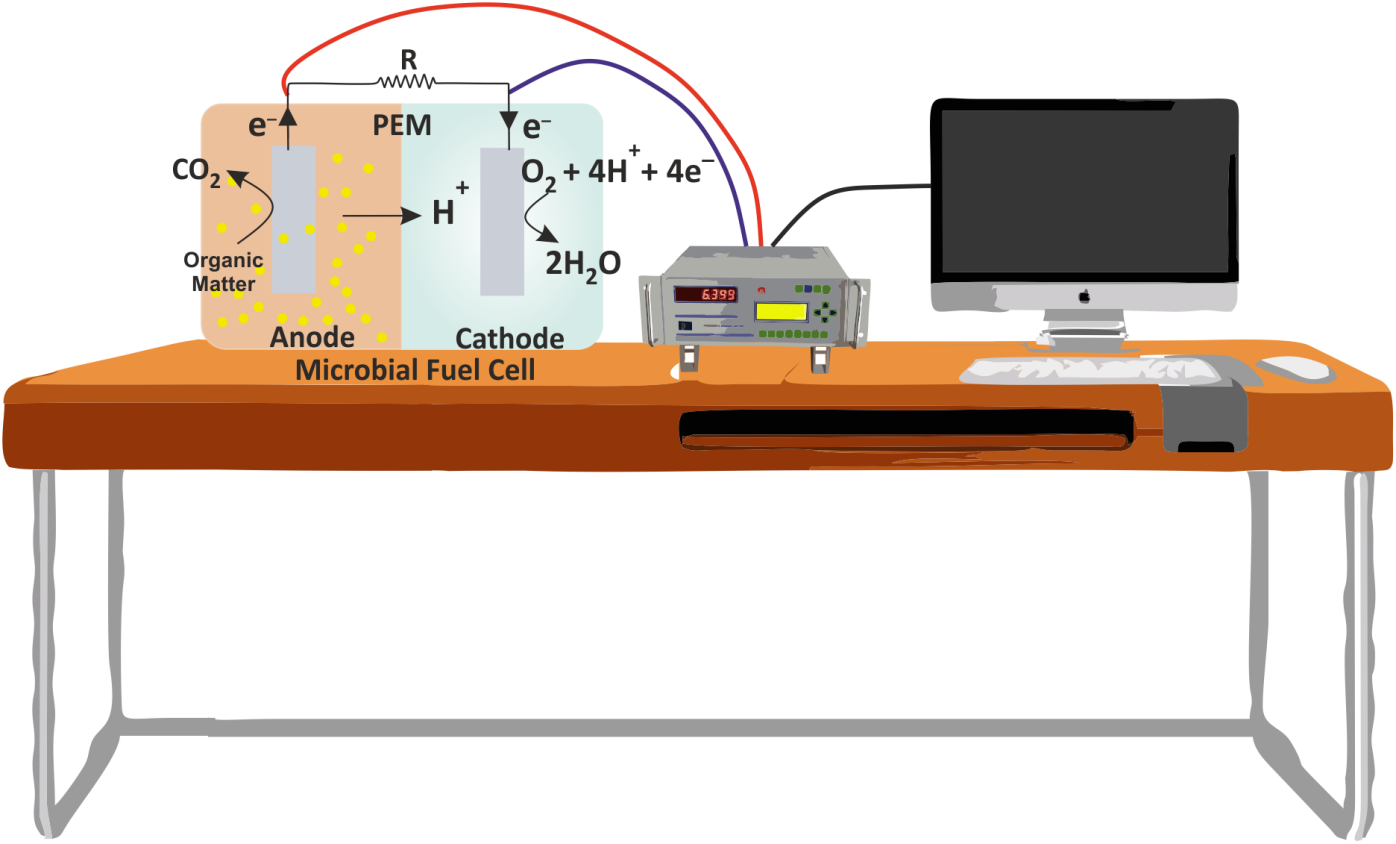
Schematic showing a typical MFC and its working principle. PEM, Proton exchange membraner and R, Resistance.

The electrigens reduce the anode by oxidizing the organic matter present in the anodic chamber. The process of anode reduction is thermodynamically favorable and hence spontaneous. The anodic redox potential is dependent upon the chemical nature of organic matter and can be calculated using the well-known Nernst equation. On the other hand, electrons in the cathodic chamber are commonly accepted by oxygen due to their availability and high redox potential (+0.82 V). Still, a number of other chemical acceptors are also used that include nitrate, manganese oxide, iron, hydrogen peroxide and nitrite (Chaudhuri and Lovley, 2003). The schematic representation of MFC is shown in **Figure 2**. The basic functional mechanisms of MFC would be clearer by an example of reactions on the electrodes surface, as shown below:

Anode:


(E_anode_ = -0.28V) (2.1) $$CH_{3}COO^{-}+2H_{2\;}O\to 2CO_{2}+7H^{+}+8e^{-}$$

Cathode:


(E_cathode_ = +0.82V) (2.2) $$O_{2}+4e^{-}+4H^{+}\to 2H_{2}O$$

The overall cell voltage can be described as:


(2.3) $$E_{cell}=E_{cathode}-E_{anode}\;\left(+1.1V\right)$$

The ideal cell voltage that a system can generate is represented by equation 2.3, but due to the association of several losses in real MFC, the operating voltage is lowered. Primarily, there are 3 integral components of a typical MFC, namely anode, cathode and proton/cation exchange membrane.

#### 4.1.1 Anode

The anode should have the following properties: (i) corrosion resistance, (ii) high electrical conductivity, (iii) biocompatibility, (iv) high surface area, (v) chemical stability and mechanical strength (Rinaldi et al., 2008; Guo et al., 2015). Carbon based conductive electrodes are frequently used in an anodic chamber. Classical examples include carbon paper, carbon brushes, carbon felt, reticulated vitreous carbon, graphite fiber brush, granular graphite, graphite plates, and rods (Zhou et al., 2011; Hindatu et al., 2017).

#### 4.1.2 Cathode

The MFC cathode can be biotic or abiotic. Carbon based electrodes are the most preferred choice as a cathode as well. The abiotic electrodes generally have chemical/metal catalysts for acceptor reduction. Biotic electrodes, on the other hand, have algae/bacteria which aid both in acceptor reduction and production. Platinum based electrodes find applications in chemical fuel cells for high efficiency oxygen reduction at the cathode (Liu et al., 2004). Pt based electrodes are not suitable for biotic cathodes because of several reasons. Pt is poisoned by phosphates, nitrates, and chlorides often used in the microbial growth medium. Pt is costly and also toxic to microorganisms (Khandelwal et al., 2021). Non-platinum based catalysts like carbon nanotube, conductive polyaniline, metal oxide (lead oxide- PbO_2_, manganese (IV) dioxide), metals (cobalt and iron) serve well in biotic cathodes (He et al., 2017).

#### 4.1.3 Membrane/Separator

In order to maintain chemical equilibrium in the cell, usually, a membrane or separator is placed between the anode and cathode which ensures the protons and/or cations transport from the anodic to the cathodic chamber. **Table 3** summarizes the membranes or separators employed in MFCs. The most commonly used membrane in conventional MFCs is Nafion 117. It is resistant to biofouling, has high ionic conductivity, and is impermeable to oxygen and organic acids (Logan and Regan, 2006). Its employability is limited by its high cost. In addition to this, researchers have used glass fibers, J-cloth, earthenware, nylon fibers, ceramics, and biodegradable shopping bags as alternative membrane separators (Santoro et al., 2017).

**Table 3:** Different separators used in MFCs and their performances.

Membrane/Separator	Base material	Proton conductivity	Power density (mW/m^2^)	Reference
SPEEK	Sulfonated poly (ether ether ketone)	0.163 x 10^-2^	77	Narayanaswamy Venkatesan and Dharmalingam (2015)
SPEEK/GO	SPEEK/Grapheme oxide composite	2.55 x 10^-3^	41.70	Shabani et al. (2020)
PES/SPEEK	Sulfonated poly (ether ether ketone)/poly (ether sulfone)	2.56 x 10^-5^	170	Lim et al. (2012)
SPEEK/SiO_2_	SPEEK/SiO_2_	1.018 x 10^-2^	1008	Sivsankaran and Sangeetha (2015)
SPEEK/TiO_2_	SPEEK/TiO_2_ composite	0.187 x 10^-2^	98.1	Narayanaswamy Venkatesan and Dharmalingam (2015)
SPES/PES	Sulfonated polyether sulfone/polyether sulfone	–	59	Zinadini et al. (2017)
PS/SPEEK	(Polysulfone)/(sulfonated poly ether ether ketone)	–	97.47	Ghasemi et al. (2016)
Nafion-112	Perfluorinated membrane	4.8 x 10^-2^	19.7	Ilbeygi et al. (2015)
Nafion-117	Perfluorinated membrane		106.7	Ghasemi et al. (2013)
PVA/Nafion/borosilicate	Polyvinyl alcohol-Nafion-borosilicate	0.07	–	Tiwari et al. (2016)
Flemion	Fluorinated membrane mfg. by Asahi Glass Company, Japan	–	200	Hosseini and Ahadzadeh (2012)
PVDF-g-PSSA	Poly (-vinylidene fluoride) grafted sodium styrene sulfonate	0.046	147	Xu et al. (2019)
UF-1kDa	Ultra filteration membrane	–	36	Hou et al. (2011)
Ceramic	Clay	–	5.23 W/m^3^	Jadhav et al. (2016)
Ceramic	Terracotta	–	400mW	Ieropoulos et al. (2016)
Ceramic	Fine fire clay	–	2.1 mW	Merino-Jimenez et al. (2017)
Ceramic	Mullite & terracotta	–	27 W/m^3^	Tremouli et al. (2018)
Clay blended with rock phosphate	Clay blended with rock phosphate	–	960 mW/m^3^	Khandelwal et al. (2020)
UF	Ultra-filtration membrane (0.45 µm)	–	6 W/m^3^	Khandelwal et al. (2021)

### 4.2 Photosynthetic or algae assisted MFCs

As mentioned earlier, algae assisted MFCs hold significant promise in making MFC technology sustainable. Algae-assisted MFCs can be powered by low-cost algae biomass; can produce algae biomass which serves the dual purpose of carbon capture, and oxygen generation. Oxygen is the most preferred electron acceptor in MFCs as it supports high potential differences. Algae cultivation at the cathode provides the system with a continuous supply of oxygen (during the light period) and helps circumvent the installation of mechanical aerators. Algae biomass also serves as feedstock for biodiesel generation and several other products. Wang et al. reported a power density of 5.6 W/m^3^ in a *Chlorella* based MFC (Wang et al., 2010). A culture of cyanobacteria, *Anabaena*, at the cathode sparged with a CO_2_-air mixture gave a power density of 57.8 mW/m^2^ (Pandit et al., 2012). In a study, a power density of 2.48 W/m^3^ and a Coulombic efficiency (CE) of 9.4% were attained using immobilized algae systems (Zhou et al., 2012). Photosynthetic microbial fuel cell (PMFC), algae assisted microbial fuel cell (AMFC) or microbial carbon capture cell (MCCs) also serve as a modified photo bioreactor equipped with an inherent oxygen quenching mechanism and carbon dioxide supply. The process of algae cultivation at the cathode also complements the effective carbon removal at the anode. Microalgae biomass is rich in hydrolysable carbohydrates, fats, and proteins and can serve as an electron donor substrate at the anode (Cui et al., 2014).

**Figure 3:**
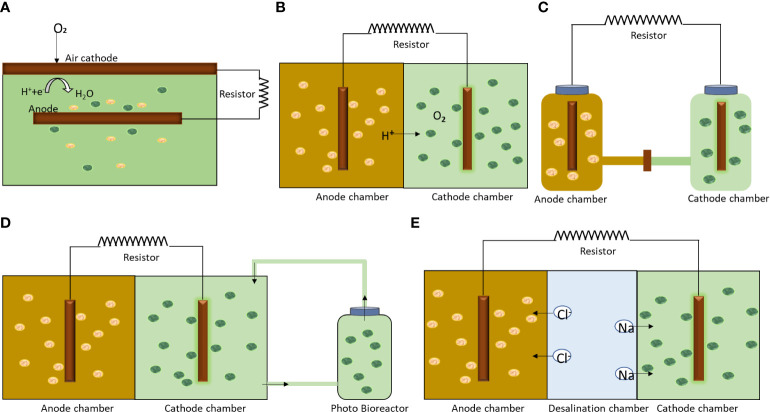
Schematic showing different algae assisted MFC configurations **(A)** single chamber; **(B)** Dual chamber; **(C)** H-shaped; **(D)** dual chamber integrated with external photobioreactor and **(E)** three chamber with desalination.

An algae assisted MFC can take different configurations depending on the intended application, i.e., algal production, power generation and wastewater treatment. Various kinds of algae-based MFC configurations are shown in **Figure 3**. The configuration varies from triple chamber to single chamber. A single chamber algae assisted MFC involves bacterial and algae cultivation in the same chamber. The CO_2_ produced by bacteria is effectively sequestered by the microalgae present in the same chamber. The carbon capture is efficient and the system is easily maintainable. In a two chambered system, algae and bacterial consortia are separated by a proton exchange membrane (PEM). These systems are used for algae cultivation for bioenergy or other applications. A separate photo-bioreactor is sometimes coupled with the system to enhance the algae growth rate and power generation. A three chamber algae-based MFC finds application in water desalination, where saltwater is fed to the middle chamber to facilitate flow of positive and negative ions. Researchers have also used uplift aeration type MFC to support high algae growth rates (Saba et al., 2017).

## 5 Use of algae in MFCs

### 5.1 Algae biomass as anodic substrate

Algae biomass is rich in decomposable carbohydrates, lipids and proteins. Therefore, algae serve as a good source of electron donor at the anode. The primary challenge with the use of intact algae biomass is its complex cell wall. Algae cell wall composition varies from class to class and species to species. The chlorophycophyta contain a wide array of cell walls ranging from cellulose pectin complexes to hydroxyproline rich glycoproteins. Like plants cells, algae cell walls are intricate mix of polymers such as cellulose, hemicellulose, lignin, pectin and arabinogalactan proteins. This complex assembly of polymers in the algae cell wall necessitates the biomass pretreatment to break open the structure, enhance the surface area, and hydrolyze polymers. Researchers have used both intact and pretreated micro and macroalgae as anodic substrates and reported good power outputs (Velasquez-orta et al., 2009; Cui et al., 2014). The use of pre-digested algae biomass also supports high power output over undigested biomass (Salar-garcía et al., 2016).

### 5.2 Algae biomass at the cathodic chamber

Algae at the cathode not only serve as oxygen supplier but also as catalysts for oxygen reduction at the electrode surface. Algae produced metabolites also serve as electron acceptors in the absence of oxygen particularly during the dark period. The success of algae assisted MFC depends on the process of photosynthesis which is driven by light energy and carbon dioxide supply (González Del Campo et al., 2013; Elmekawy et al., 2014).

Additionally, algae can effectively remove nitrates and phosphates from the water. Algae can grow in autotrophic, heterotrophic, and mixotrophic mode. Heterotrophic and mixotrophic modes assist with carbon removal. The simultaneous carbon, nitrogen, and phosphorus removal is possible using dual chamber algae assisted MFCs. The anode and cathode both can contribute towards carbon removal while the algae assisted cathode can help with nitrogen and phosphorus removal (Commault et al., 2017). The success of algae assisted MFC in wastewater treatment depends on the algal strain, inoculum size, density, temperature, N/P ratio, salinity, pH, light intensity and CO_2_ supply and capture rate. An algae assisted MFC thus needs optimization with respect to all these parameters (Nagendranatha Reddy et al. 2019).

## 6 Output from algae assisted MFC

Algae assisted MFCs can generate both bioelectricity and algal biomass. **Table 4** summarizes the prominent studies in terms of power output obtained from different algal strains employed in algae assisted MFCs. Power output from algae assisted MFC can be optimized by choosing an appropriate algae species, electrode material, catalyst coating, chamber design, light duration and intensity, electron donor substrate, and CO_2_ source. The microalgae can directly generate current either by introducing it in the anodic chamber as an electron donor substrate or in the cathodic chamber as a biocatalyst for generation of oxygen (Elmekawy et al., 2014).

**Table 4:** Different species of microalgae and the corresponding dissolved oxygen (DO) and power output obtained in algae assisted MFCs.

Reactor configuration	Algae species	DO concentration (mg/L)	Power Density	Reference
Dual-chamber	*Chlorella*	–	3720 mW/m^3^	Zhang et al. (2019)
Single chamber	*Scenedesmus quadricauda*	–	62.93 mW/m^2^	Yang et al. (2018)
Dual- chamber	*Mix culture*	19.57	50 mW/m^2^	Nguyen et al. (2017)
Sediment MFC	*Mix culture*	14.2	22.19 mW/m^2^	Neethu and Ghangrekar (2017)
Tubular	*Chlorella*	–	200 mA/m^2^	Ma et al. (2017)
Two chamber	*Spirulina*	–	0.85 W/m^2^	Colombo et al. (2017)
Air-lift type MFC	*C. vulgaris*	5.65	558 mW/m^3^	Hu et al. (2016)
Two chamber	Mix culture	–	128 µW	Gajda et al. (2015)
Two chamber	*C. vulgaris*	–	34.2 mW/m^2^	Commault et al. (2017)
Two chamber	*C. vulgaris*	8.5	126 mW/m^3^	Bazdar et al. (2018)
Two chamber	*C. vulgaris*	–	1926 mW/m^2^	Cui et al. (2014)
Two chamber	*Microcystis aeruginosa*	–	58.4 mW/m^3^	Cai et al. (2013)
Two chamber	Mix culture	20.8	0.35 V	Kakarla and Min (2019)
Two chamber	*C. vulgaris*	12	14.40 mW/m^2^	González et al. (2013)
Two chamber	*C. vulgaris*	100% saturated DO in water	23.97 mW/m^2^	Gonzalez del Campo et al. (2014)
Two chamber	*C. vulgaris*	7	42.98 mW/m^2^	Gonzalez Del Campo et al. (2015)
Two chamber	*C. vulgaris*	–	62.7 mW/m^2^	Gouveia et al. (2014)
Two chamber	*Chlorella* sp. *QB-102*	–	36.4 mW/m^2^	Zhang et al. (2018)
Two chamber	*C. vulgaris*	–	327.67 mW/m^2^	Huarachi-Olivera et al. (2018)
Two chamber	*Synechococcus* sp.	10.2	110.92 mW/m^2^	Lakshmidevi et al. (2020)
Two chamber	Chlorella sp. G29-5	–	505.6 mW/m^2^	Wu et al. (2021)
Two chamber	*Cladophora* sp.	18.7	619.1 mW/m^2^	Taşkan and Taşkan (2022)
Two chamber	*Dunaliella salina*	5.83	213.38 mW/m^2^	Mishra and Chhabra (2022)

The algal biomass production in algae assisted MFC is important to assess the overall system performance and net energy recovery. The **Table 5** summarizes the key studies reporting the chemical oxygen demand (COD) removal by particular algal strains and their net biomass production. The main factors which affect the algal growth in MFC include reactor configuration, wastewater composition and light intensity (Luo et al., 2017). Despite some obvious advantages, the algae growth rates and productivities achieved in MFCs are low. This is primarily due to the lack of studies specifically investigating the algae growth rate in MFCs and on system scale up.

**Table 5:** COD removal and algal biomass generation in algae assisted MFCs.

MFC type	Substrate or nutrient media used in cathode	Algal strain	Removal efficiency (%)			Biomass concentration (mg/l)	Reference
			COD	TN	TP		
Double chamber	Synthetic media	*Chlorella* sp. *QB-102*	–	–	–	–	Zhang et al. (2018)
Double chamber	Landfill leachate wastewater	Mixed culture	52.8	80	–	–	Nguyen et al. (2017)
Double chamber	Synthetic media	Mixed culture		100			Kakarla and Min (2019)
Dual chamber integrated with photobio- reactor	CO_2_	Mixed culture				470	Gajda et al. (2015)
Dual chamber	CO_2_	*Chlorella vulgaris*	80			360	González et al. (2013)
Single chamber	CO_2_	*Chlorella vulgaris*	44			270	Hou et al. (2016)
Dual chamber	Chocolate factory	*Chlorella vulgaris*	78.6			5.2	Huarachi-Olivera et al. (2018)
Single & dual chamber	Synthetic media	*Spirulina*	89	5.5	17		Colombo et al. (2017)
Dual chamber	Anodic effluent	*Chlorella vulgaris*	49	83			Commault et al. (2017)
Dual chamber	CO_2_ from anode chamber	*Chlorella vulgaris*	90			1247	Cui et al. (2014)
Dual chamber	Externally supplied CO_2_	*Chlorella vulgaris*	5.5			3600	Bazdar et al. (2018)
Dual chamber	CO_2_ from anode chamber	*Scenedesmus acutus PUVW12*	87			290	Angioni et al. (2018)
Single chamber	Anaerobically digested kitchen waste effluent	*Golenkinia* sp. *SDEC-16, Scenedesmus* *SDEC-8 & Scenedesmus* *SDEC-13*	43.6	38	100	325	Hou et al. (2016)
Two chamber	municipal solid waste leachate	*Synechococcus* sp.	76.5	90.2	94.3	254	Lakshmidevi et al. (2020)
Two chamber	Synthetic wastewater	*Dunaliella salina*	59.32	–	–	4.02 ± 6 × 10^6^ cells/ml	Mishra and Chhabra (2022)

## 7 Factors affecting power output in algae assisted MFC

### 7.1 Light

Light is the primary requirement for photosynthesis. Light intensity, its duration (light/dark period), and wavelength all affect algae growth. High light intensities lead to photo oxidation and growth inhibition. On the other hand, low light intensities lower algae growth rates and promote bacterial growth. Both polychromatic and monochromatic light is used for cultivating algae. Amongst the monochromatic light, the red and blue light is most preferred for high rate algal cultures. Light source and its orientation with respect to MFC affect algae growth. Researchers prefer using inbuilt LED lights that ensure direct illumination and minimize the self-shading effect. It also helps to regulate temperature and ensure low temperatures enabling optimized algae growth. The ratio of light/dark period is also critical and varies from species to species and system to system (Saba et al., 2017; Nagendranatha Reddy et al. 2019).

### 7.2 CO_2_ concentration

Carbon dioxide is another key ingredient required for algae growth. Most of the algae grow well at atmospheric CO_2_ levels. However, higher concentrations are shown to promote algae growth and carbon capture (Singh and Singh, 2014). Researchers have studied the impact CO_2_ concentration on growth and lipid production in algae (Sato et al., 2003; Wang et al., 2010). The response varies from species to species and also on the cultivation conditions. In an algae assisted MFC, the CO_2_ required by the microalgae can either be the CO_2_ present in the anodic off-gas (Wang et al., 2010) or it can be CO_2_ sparged separately (González Del Campo et al., 2013). However, CO_2_ sparging is associated with certain disadvantages, including the lowering of the pH on the dissolution of CO_2_ in water, which can be resolved by the use of a higher initial inoculum concentration (Chiu et al., 2009; Zhang et al., 2014). Increasing the CO_2_ concentration by 10-15% has resulted in a 6% increase in the lipid content, confirming the significance of the CO_2_ concentration in the lipid content of algal cells (Liu et al., 2011).

### 7.3 Dissolved oxygen

Photosynthesis liberates oxygen *via* light reaction and algae consume oxygen while respiring. A high concentration of oxygen becomes inhibitory for algae growth and leads to photo-oxidative damage. It was found that a DO concentration exceeding 30 mg/l inhibited the *C. vulgaris* growth by 30% (Kazbar et al., 2019). MFC circumvents this problem as oxygen is quenched through the reduction reaction in a circuit MFC. The solubility of oxygen in water is also dependent on temperature, salt content, and duration of light/dark cycles. It is often observed that during night time, the DO level drops and so is the power output from a MFC (Gonzalez Del Campo et al., 2015). A DO level of 4.5-5.5 mg/l is suitable for supporting continuous power output from a MFC (Rodrigo et al., 2010), while an algae based cathode can realize DO levels in the order of 6.6 mg/l (Kang et al., 2003). Another major factor that determines the DO concentration in water is temperature. Hence, the use of proper lighting equipment with useful wavelengths is of utmost importance. In other words, DO is dependent on the temperature as well as the duration of the light/dark cycles. One important investigation to show this relationship was the one carried out by Gouveia et al. (2014) wherein experiments were conducted using two light intensities 26 µE/m^2^ and 96 µE/m^2^. An increase in algal growth rate with increased oxygen concentration resulting in enhanced power generation at higher light intensity was observed in this investigation (Gouveia et al., 2014).

## 8 Scaled-up studies on algae-assisted MFCs

The employment of microalgae in the cathodic chamber of MFC is comparatively a new technology, therefore, there are thus far not so many large-scale studies involving AMFCs. However, there are numerous reports for MFC applications on larger scale for wastewater treatment and bioelectricity generation. Scaled-up implementation of MFCs can be done either by increasing the size of a single reactor or stacking several miniature reactors. However, miniaturization does not increase internal resistance much, hence this strategy can provide uncompromised power output. In a recent study, a low-cost liter scale AMFC was constructed using inexpensive materials such as rock phosphate (RP) blended clayware as anodic and low-density polythene bag as cathodic chamber. This study was carried out under natural sunlight in outdoor conditions without controlling temperature and pH. The slow release of P from RP resulted in enhanced algal growth of 4.6 g/l along with a power density of 1.2 W/m^3^. This inexpensive AMFC assembly costs only 11.25 USD, implying the possibility of large-scale application (Khandelwal et al., 2020). In an another study, multiple anodes were connected in series with capacitors and this stacked assembly was operated in an algal raceway pond (16 L). The highest voltage and power output obtained from this study was 1.4 V and 2.34 W/m^3^, respectively (Yang et al., 2019).

Recently, sediment microbial fuel cells (SMFCs) have also emerged as a novel technology to treat sedimental wastes. Sharma et al. (2021), compared two SMFCs, having plant and microalgae in the cathode chamber. The algae-based SMFC performed better in terms of COD, phosphate and nitrate removal and the algae biomass productivity of 0.031 Kg/m^3^/d was attained (Sharma et al., 2021). Similarly, in another study involving SMFC, a power density of 5.17 W/m^3^ was obtained using *Chlorella vulgaris* at the cathode (Song et al., 2020).

In addition to this, most of the AMFC studies have been carried out in batch mode, whereas to bring the technology from lab to the real world the process should be either in continuous or semi-continuous mode (Kannan and Donnellan, 2021). Nguyen and Min (2020) treated leachate wastewater in continuous mode with a hydraulic retention time of 20 h. The effluent from anodic compartment was recycled to the cathodic chamber for the growth of algae, ensuring maximum removal of COD and reutilization of nutrients (Nguyen and Min, 2020).

## 9 Future outlook

This review summarizes the potential of third generation biofuels and their integration with BESs. Several studies have shown the possible applications of AMFCs at commercial scale. Researchers have explored few macroalgae spp. in AMFCs and concluded that employment of macroalgae in AMFCs can be more beneficial than that of microalgae in terms of waste treatment and algal biomass harvesting. Further research is required to assess the full potential of AFMCs. In addition, approaches to increase the efficiency by selecting the correct microbial consortia/microalgae, required for wastewater treatment, biofuel, biomass and bioelectricity generation are of paramount importance.

Considerable research has also been carried out on genetical modification of microalgae to magnify their cellular potential. These genetic engineering strategies along with omics would extend the existing knowledge of metabolic pathways. However, studies on employment of genetically modified algae in AMFCs are very limited and need further investigation.

The better understanding of the electron transfer mechanisms between electrode and microbes can further aid in selecting suitable strains and electrode materials to boost the power output. Additionally, the selection of better membranes and electrodes which are easily scalable at affordable cost needs further research. Also, novel engineering solutions for reactor design to promote algal growth and boost power output along with easy harvesting of algal biomass, are required. Furthermore, integration of AMFCs with different technologies such as anaerobic digestion (AD) can add more value to the overall operation and increase process efficiency, nutrient uptake and waste removal. For example, a typical AD process treating food waste can generate biomethane and the AD effluent can be utilized in AMFCs for both bacteria and algae in the anodic and cathodic chamber, respectively. The overall output from this coupled process such as biomethane, bioelectricity, waste treatment and algal biomass can be developed as a biorefinery.

## Author contributions

AK: Conceptualization, Writing - original draft. MC: Conceptualization, Writing - review and editing, Visualization, Supervision. PL: Writing - review and editing, Supervision, Resources. All authors contributed to the article and approved the submitted version.
